# The Influence of Three Modes of Human Support on Attrition and Adherence to a Web- and Mobile App–Based Mental Health Promotion Intervention in a Nonclinical Cohort: Randomized Comparative Study

**DOI:** 10.2196/19945

**Published:** 2020-09-29

**Authors:** Melanie Elise Renfrew, Darren Peter Morton, Jason Kyle Morton, Jason Scott Hinze, Geraldine Przybylko, Bevan Adrian Craig

**Affiliations:** 1 Lifestyle and Health Research Centre Avondale University College Cooranbong Australia

**Keywords:** human support, adherence, attrition, engagement, web-based mental health, health promotion, eHealth, SMS, videoconferencing

## Abstract

**Background:**

The escalating prevalence of mental health disorders necessitates a greater focus on web- and mobile app–based mental health promotion initiatives for nonclinical groups. However, knowledge is scant regarding the influence of human support on attrition and adherence and participant preferences for support in nonclinical settings.

**Objective:**

This study aimed to compare the influence of 3 modes of human support on attrition and adherence to a digital mental health intervention for a nonclinical cohort. It evaluated user preferences for support and assessed whether adherence and outcomes were enhanced when participants received their preferred support mode.

**Methods:**

Subjects participated in a 10-week digital mental health promotion intervention and were randomized into 3 comparative groups: standard group with automated emails (S), standard plus personalized SMS (S+pSMS), and standard plus weekly videoconferencing support (S+VCS). Adherence was measured by the number of video lessons viewed, points achieved for weekly experiential challenge activities, and the total number of weeks that participants recorded a score for challenges. In the postquestionnaire, participants ranked their preferred human support mode from 1 to 4 (S, S+pSMS, S+VCS, S+pSMS & VCS combined). Stratified analysis was conducted for those who received their first preference. Preintervention and postintervention questionnaires assessed well-being measures (ie, mental health, vitality, depression, anxiety, stress, life satisfaction, and flourishing).

**Results:**

Interested individuals (N=605) enrolled on a website and were randomized into 3 groups (S, n=201; S+pSMS, n=202; S+VCS, n=201). Prior to completing the prequestionnaire, a total of 24.3% (147/605) dropped out. Dropout attrition between groups was significantly different (*P*=.009): 21.9% (44/201) withdrew from the S group, 19.3% (39/202) from the S+pSMS group, and 31.6% (64/202) from the S+VCS group. The remaining 75.7% (458/605) registered and completed the prequestionnaire (S, n=157; S+pSMS, n=163; S+VCS, n=138). Of the registered participants, 30.1% (138/458) failed to complete the postquestionnaire (S, n=54; S+pSMS, n=49; S+VCS, n=35), but there were no between-group differences (*P*=.24). For the 69.9% (320/458; S, n=103; S+pSMS, n=114; S+VCS, n=103) who completed the postquestionnaire, no between-group differences in adherence were observed for mean number of videos watched (*P*=.42); mean challenge scores recorded (*P*=.71); or the number of weeks that challenge scores were logged (*P*=.66). A total of 56 participants (17.5%, 56/320) received their first preference in human support (S, n=22; S+pSMS, n=26; S+VCS, n=8). No differences were observed between those who received their first preference and those who did not with regard to video adherence (*P*=.91); challenge score adherence (*P*=.27); or any of the well-being measures including, mental health (*P*=.86), vitality (*P*=.98), depression (*P*=.09), anxiety (*P*=.64), stress (*P*=.55), life satisfaction (*P*=.50), and flourishing (*P*=.47).

**Conclusions:**

Early dropout attrition may have been influenced by dissatisfaction with the allocated support mode. Human support mode did not impact adherence to the intervention, and receiving the preferred support style did not result in greater adherence or better outcomes.

**Trial Registration:**

Australian New Zealand Clinical Trials Registry (ANZCTR): 12619001009101; http://www.anzctr.org.au/ACTRN12619001009101.aspx

## Introduction

The burden of mental distress is pervasive globally and includes common mental health disorders such as depression and anxiety. Approximately 300 million people worldwide are affected by depression—the principal cause of global disability [1]. Depression is frequently comorbid with other diseases and severely compromises effective functioning for individuals, negatively impacting family and work environments [1]. Furthermore, indicators suggest that even the general population is increasingly experiencing mental distress, including severe stress, anxiety, depressive symptoms, a sense of isolation, and feeling overwhelmed [2,3].

While a growing repertoire of digital interventions is improving accessibility to treatment options for people with common mental health disorders, there is also an urgent need for easily accessible mental health promotion interventions (MHPIs) to improve the mental well-being of nonclinical population groups. MHPIs that focus on enhancing psychological well-being may provide an important buffer against mental distress, potentially attenuating the mental health burden. Furthermore, lifestyle-focused MHPIs might also ameliorate symptoms for those who have already been diagnosed with a common mental health disorder [4].

Innovative web- and mobile app–based technologies allow MHPIs to be disseminated widely and cost effectively to maximize accessibility. However, despite the many advantages of digital interventions, both high dropout attrition (ie, participants who drop out early or who are lost to follow-up) and nonusage attrition (ie, nonadherence) are persistent problems [5-7].

In his formative publication titled “The Law of Attrition,” Eysenbach [8] called for the methodical study of attrition in eHealth interventions because, unlike drug trials, it is usually easy for participants to both join and withdraw from a digital intervention, especially when they are not critical to life—sometimes described as “easy-in” and “easy-out” [9]. Maximizing adherence, defined as “the degree to which the user followed the program as it was designed” [10], is a complex challenge for researchers and health care providers. Additionally, definition differences and measurement heterogeneity between studies are problematic [11-13]. Nevertheless, many factors affecting adherence have been identified.

Influences on adherence are multifactorial, and consistency in adherence patterns have been elusive [12,14]. A 2018 review of theoretical perspectives on adherence suggested the need for interdisciplinary collaboration to better understand patterns of adherence due to a diverse range of technological, environmental, and individual influences [12]. For instance, technological factors may include website design, persuasive systems design [15,16], behavior change techniques [17], human support factors [6,8,13,18,19], personalized content (ie, tailoring) [13], frequent updates and dialogue support (ie, praise, rewards, and reminders) [18], and gamification techniques [20]. Environmental influences consist of factors such as socioeconomic status, employment status, education level [21], internet or computer accessibility [22], literacy [21,23], culture, the health care system, family and community support [24], and time availability [13,22]. Examples of individual factors include whether a person self-selects into a study and invests effort [25], planning and self-efficacy [21,26], compatibility with personal values [8], motivation factors [6,22], focus on immediate benefit rather than long-term goals, perceived treatment credibility [13,27], receiving preferences [28], health status, psychological vulnerability [21-23], user expectations [6,13,23], gender [13,21], and age [17,21].

### Human Support and Adherence

#### Clinical Settings

Despite the broad range of factors listed, adherence has frequently been positively associated with human support (ie, guidance) in clinical settings. A qualitative systematic review of 64 studies reported that adherence was improved by support of counselors, peers, and phone and email contact [24]. A 2012 systematic review and meta-analysis concluded that supported interventions yielded better retention and outcomes [29]. Other randomized controlled trials (RCTs) have also reported similar links between human support and adherence [14,30], though greater adherence does not always translate to better outcomes; and adherence may be problematic for individuals with depression, irrespective of the support received [30].

A 2017 scoping review [31] analyzed 19 RCTs from 2000 to 2016 that considered human support factors in internet-based interventions for depression and anxiety. The review identified 7 different human support factors (guided vs unguided, therapist expertise, human vs automated, scheduled vs unscheduled, support mode, synchronicity, and support intensity) and analyzed them for improvement in clinical outcomes and adherence. While just one human support factor (scheduled support) was associated with significantly improved outcomes, results were mixed in relation to adherence, with human support improving adherence in only 4 out of 9 studies [31].

Recent web-based interventions for common mental health disorders, comparing supported and unsupported arms, have found that well-designed self-guided interventions achieve significant improvements in outcomes and maintain high adherence rates irrespective of support provided [32-34]. Notably, in some cases, treatment satisfaction was higher in supported arms [34,35] and some participants perceived support as necessary to success [36].

Human support requirements to encourage adherence may be vastly different in nonclinical populations compared to clinical cohorts who experience symptoms that may preclude them from engaging with an intervention. Therefore, it is vital to determine if human support adds value to an intervention for a nonclinical cohort because unsupported interventions can be administered at a lower cost and be more easily distributed in a scalable manner.

#### Nonclinical Settings

Studies evaluating the influence of human support on adherence to MHPIs among nonclinical populations are scant in comparison to clinical cohorts. A study involving a mindfulness intervention, targeting college students and young working adults, found that despite telephone or email support, adherence was poor and nonadherers had poorer mental well-being and lower energy and treatment expectancy [37]. The researchers suggested a greater need for collaboration between health professionals and information technology experts to improve the “personalization” of digital interventions to enhance adherence [37]. A Swedish study that implemented an internet-based relaxation program found that human support did not affect treatment outcomes or adherence [27,35]. Outcomes were positively associated with completing the homework (ie, behavioral tasks) but not engagement with the online aspect of the program. Early attrition was predicted by low belief in the treatment; and nonadherence was associated with increased stress symptoms, lower levels of intrinsic motivation, and a greater focus on immediate consequences of behavior as opposed to long-term gains. Conversely, adherence was predicted (positively) by education level and treatment credibility.

In a pooled analysis of 3 web-based studies [14], researchers investigated the influence of 3 types of support: content-focused (ie, personalized email feedback), adherence-focused (ie, monitoring adherence and sending reminders), and administrative support (ie, access to contact details to ask for technical assistance). Those who received content- and adherence-focused support completed more modules than those who received only administrative support. However, the researchers concluded that even after taking human support and other demographic variables into consideration, most interindividual variations in nonadherence remained largely unsolved [14]. A web-based mindfulness and stress management RCT [38] compared the effects of no support, group support only, and group support with added clinician support on engagement and outcomes. Group support improved outcomes and adherence, but extra clinician support added no benefit. Notably, although the program was web-based, support was provided face-to-face in the workplace.

We have previously reported the mental health outcomes of this study and found that no difference was observed between groups; improvements in outcomes were obtained irrespective of the differing modes of human support offered [39]. This study focused on examining attrition and adherence patterns between the groups. We also evaluated user preferences for human support and assessed whether adherence and mental health outcomes were enhanced when participants received their preferred mode of human support. The outcomes of the study contribute toward understanding the value of human support on adherence to a web- and mobile app–based MHPI targeting a nonclinical cohort. This is vital information for researchers and clinicians in order to inform the optimal delivery of digital MHPIs in a cost-effective, accessible, and scalable manner.

## Methods

This section provides a brief summary of methods used. For a detailed explanation of the methods, refer to our previous article that reported the influence of human support mode on mental well-being outcomes [39].

### Study Design

The multiarm randomized comparative study design included 3 intervention groups that varied by human support mode: standard (S), comprising fully automated emails only; standard plus personalized SMS text messaging support (S+pSMS); standard plus videoconferencing support (S+VCS).

### Recruitment and Randomization

**Figure 1:**
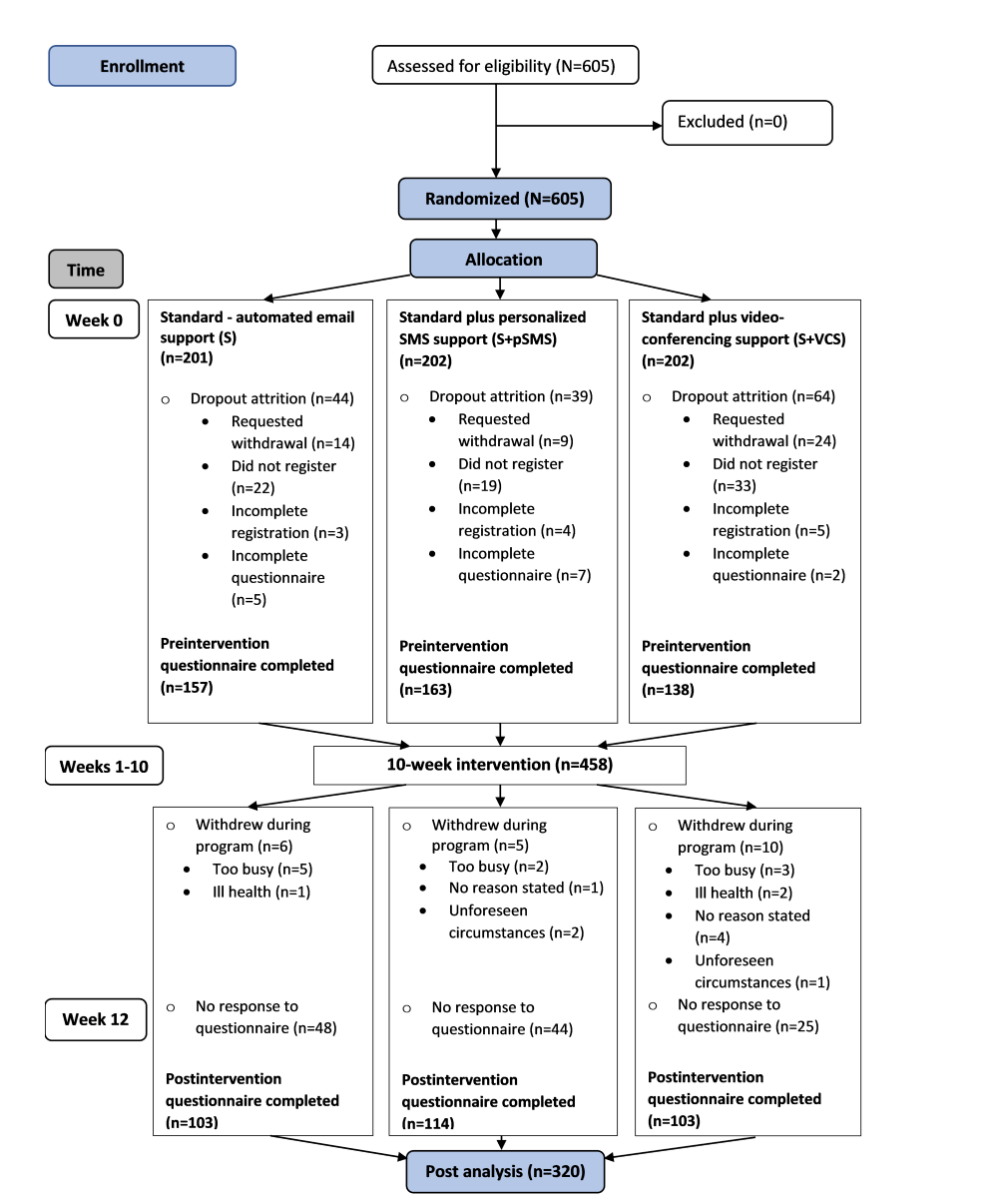
Flow of participants.

The study was advertised to members of a faith-based organization using a combination of methods (eg, email, social media, bulletins, and magazines), and interested individuals were directed to a website to apply. After randomization, eligible participants were emailed their group allocation, a participant information document, and instructions on how to complete registration onto the electronic learning management system (eLMS), signaling consent. Figure 1 demonstrates the flow of participants through the study.

### Intervention

The 10-week interdisciplinary intervention introduced participants to a range of evidence-based strategies for enhancing mental well-being, from the disciplines of lifestyle medicine and positive psychology. Participants logged onto the eLMS or mobile app to access the intervention, which included video content and a place to log daily and weekly experiential challenges (see screenshots in Multimedia Appendix 1). The website and mobile app were designed using a range of persuasive system design principles to increase engagement [15]. Each week, participants were encouraged to view one lesson, perform small daily challenges, and complete 1 larger weekly challenge. A detailed week-by-week summary of the content and experiential challenge activities can be found in Multimedia Appendix 2.

### Human Support

The study was structured to compare the value of increasingly greater levels of human support on adherence. Principles of the adherence-focused supportive accountability model [6] underpinned interactions between the support coach and participants. As the lowest form of support, one automated email was sent to the participants each week, consisting of the following: their first name as a salutation, a couple of sentences to encourage them to view the content, and a link to an introductory video by the presenter. A reminder email was sent if a participant had not logged experiential activity for 3 days, or viewed video content for 8 days. The second level of support involved SMS messages that were written and sent from a support coach who focused on ensuring content was process-focused; messages were sent regularly, and tone exuded positivity. Messages addressed the recipient by first name and were signed by the support coach. They were sent 3 times weekly for the first 3 weeks, and twice weekly for the remaining 7 weeks. In the highest level of support, the opportunity to develop key tenets of the supportive accountability model (eg, bond and legitimacy) was provided through weekly videoconferencing sessions (9 weekly time slots). Focus was given to facilitating a supportive, respectful environment where participants could share experiences safely. The facilitator sought to build trust through benevolent engagement with participants.

### Measurements

#### Dropout Attrition

Dropout attrition was measured as the total number of randomized participants who did not complete either the preintervention (ie, early dropout) or the postintervention (ie, lost to follow-up) questionnaire.

#### Adherence Measures

##### Primary Adherence—Videos Viewed

Primary adherence was measured as the total number of weekly videos viewed, out of a total of 10. A video was considered “viewed” when at least 80% of the presentation had been played.

##### Secondary Adherence—Experiential Challenge Activities

Participants were encouraged to accumulate points through daily and weekly challenge activities that involved putting the new learning into practice. Engagement with challenges was measured in 2 ways—the total weekly challenge score and the total number of weeks out of 10 in which an engagement with a challenge was recorded. Each daily challenge was worth 10 points (ie, a total of 70 points weekly), and weekly challenges were worth 30 points. Therefore, participants could score a maximum of 100 points each week throughout the 10-week intervention, thereby accumulating a total of 1000 points to be considered fully adherent for the experiential challenge activities.

##### Videoconference Adherence

Attendance records were kept for the videoconference support sessions for those in the S+VCS group. Each participant was invited to attend 1 session each week, out of a possible 9 available time slots. Participants were able to receive a maximum adherence score of 10 over the 10-week intervention.

#### Well-Being Measures

A self-report questionnaire termed the “7 Dimensions of Wellness Index” was completed by all participants on the web-based eLMS or on the mobile app, at preintervention (Week 0) and postintervention (Week 12) (Figure 1). Demographic and lifestyle-associated questions were combined with freely accessible validated instruments to measure the participant’s well-being in several domains: physical, emotional, social, vocational, intellectual, spiritual, and environmental. Validated instruments utilized for this study on mental well-being included 2 subdomains from the 36-item Short Form Health Survey (SF-36) (ie, mental health and vitality) [40]; the 21-question Depression Anxiety and Stress Scales (DASS-21) to measure depression, anxiety, and stress [41]; the Diener flourishing scale [42]; and the Diener Satisfaction With Life Scale (SWLS) [43,44]. Well-being measures are reported on in detail in the aforementioned article [39].

#### Preferences

Participants rated 4 different human support options from most preferred to least preferred (1=most preferred, 4=least preferred) as part of the postintervention questionnaire. Support options were as follows: standard (automated email only), standard plus SMS only, standard plus VCS only, and standard plus SMS and VCS combined (not offered in the study). Stratified analyses were conducted to measure adherence and outcomes for participants who received their desired human support preference.

### Statistical Analysis

Analyses were conducted using SPSS, version 25 (IBM Corp). Preintervention-to-postintervention changes were calculated using paired *t* tests. Descriptive statistics, involving means and standard deviations, measured patterns of adherence. Analysis of variance (ANOVA) was used to compare adherence between groups. Fisher exact test was utilized to analyze relationships between categorical variables, and Cohen *d* measured effect size.

### Ethics and Informed Consent

Avondale Human Research Ethics Committee granted ethics approval for the study (Approval No. 2018.09), and it was registered with the Australian and New Zealand Clinical Trial Registry (ANZCTR12619001009101). An email containing an “Information Statement” and a “Participant Consent Form” was sent to all prospective participants. The email explained that choosing to register on the eLMS would signify informed consent.

## Results

### Participants

Potential participants (N=605) enrolled on the information website and agreed to participate in the study irrespective of randomization allocation. Subjects were randomized into 3 arms (S, n=201; S+pSMS, n=202; and S+VCS, n=202) (Figure 1). During a 1-week period, after being notified of group allocation, and before the intervention commenced, 24.3% (147/605) of participants dropped out. Early dropout attrition between groups was significantly different (*P*=.009)*.* A total of 21.9% (44/201) withdrew from the S group, 19.3% (39/202) from the S+pSMS group, and 31.6% (64/202) from the S+VCS group. Table 1 shows that dropout was significantly different between groups when the S+VCS group was compared; differences were not significant between the S and S+pSMS groups.

A total of 75.7% (458/605) of randomized participants registered on the eLMS and completed the initial questionnaire (S, n=157; S+pSMS, n=163; S+VCS, n=138). Notably, there were no significant differences between the groups in age (*P*=.19), gender (*P*=.82), education (*P*=.16), or ethnicity (*P*=.34). Of the registered participants, 69.9% (320/458) completed the postintervention questionnaire (S, n=103; S+pSMS, n=114; S+VCS, n=103), resulting in 30.1% (138/458) being lost to follow-up; there was no difference between groups (*P*=.24).

**Table 1:** Dropout attrition between groups.

Group comparison	*P* value
All groups	.009
S^a^ and S+VCS^b^	.03
S+pSMS^c^ and S+VCS	.004
S and S+pSMS	.52

^a^S: standard (automated emails only).

^b^S+VCS: standard plus videoconferencing support.

^c^S+pSMS: standard plus personalized SMS.

### Adherence

#### Primary Adherence—Videos Viewed

As shown in Table 2, the number of videos viewed was not significantly different between the groups (*P*=.42)*.* Almost half of all participants in each group were fully adherent (ie, watched all 10 video sessions), with less than 10% of individuals in each group viewing no videos. Furthermore, the percentage of participants at any level of adherence did not differ significantly between groups.

**Table 2:** Primary adherence percentages and between-group comparisons.

Videos viewed		S^a^ (n=103)	S+pSMS^b^ (n=114)	S+VCS^c^ (n=103)	Between-group difference *P* value
**Number of videos viewed, n (%)**					
	10	47 (44.6)	54 (47.4)	50 (48.5)	.58
	8-9	4 (3.8)	6 (5.3)	5 (4.9)	.38
	5-7	12 (11.6)	15 (13.2)	18 (17.5)	.54
	1-4	33 (32.0)	31 (27.2)	21 (20.4)	.55
	0	7 (6.8)	8 (7.0)	9 (8.7)	.95
Videos viewed, mean (SD)		6.0 (4.04)	6.5 (3.87)	6.8 (3.75)	.42

^a^S: standard (automated emails only).

^b^S+pSMS: standard plus personalized SMS.

^c^S+VCS: standard plus videoconferencing support.

#### Secondary Adherence—Challenges

No significant differences were recorded between the groups in the mean challenge points scored (*P*=.71*)* or in the mean number of weeks in which challenge scores were recorded *(P*=.66) (Table 3). There was an overall lack of adherence to experiential challenges, as indicated by mean challenge scores less than 400 out of a possible 1000 points. Additionally, challenge scores were logged in fewer than half of the weeks for the 10-week intervention.

**Figure 2:**
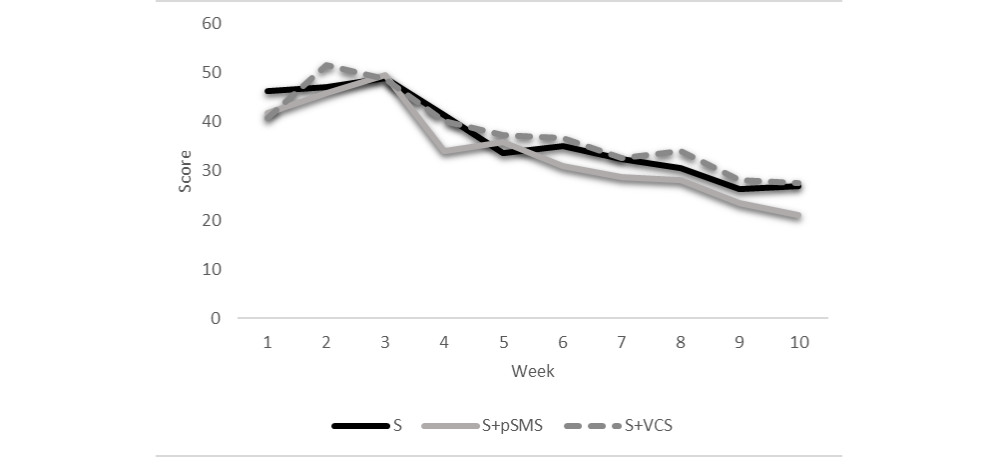
Group-based mean challenge scores over 10 weeks. S: standard—automated emails; S+pSMS: standard plus personalized SMS; S+VCS: standard plus videoconferencing support.

**Figure 3:**
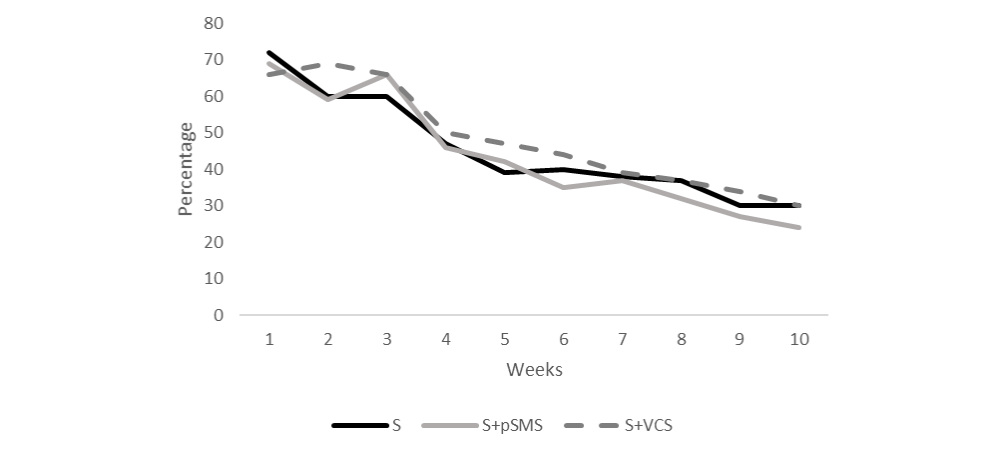
Percentage of participants who logged challenges over 10 weeks. S: standard—automated emails; S+pSMS: standard plus personalized SMS; S+VCS: standard plus videoconferencing support.

Figure 2 shows weekly mean challenge scores by group, and Figure 3 portrays what percentage of participants were logging challenges each week over the 10-week intervention. The mean number of challenge points scored was greatest in the first 3 weeks for the 3 groups. Additionally, scores declined in a graded manner from weeks 3 to 10 in each group, as illustrated in Figure 2. During the first 3 weeks of the intervention, between 59%-72% (67 to 74) participants in each group were logging challenges. The number of participants logging challenges was greatest in week 1 for the S group, highest in week 2 for the S+VCS group, and highest in week 3 for the S+pSMS group, although between-group differences were not significant. Figure 3 illustrates the steady decline in participants logging challenges from lessons 3 to 10 for all groups.

**Table 3:** Secondary adherence scores for experiential challenge activities.

Challenge activities	S^a^ (n=103)	S+pSMS^b^ (n=114)	S+VCS^c^ (n=103)	*P* value
Challenge points (out of 1000), mean (SD)	368.7 (361.6)	340.2 (339.0)	377.5 (354.0)	.71
Number of weeks in which challenge scores were logged (out of 10), mean (SD)	4.5 (3.7)	4.4 (3.4)	4.8 (3.6)	.66

^a^S: standard (automated emails only).

^b^S+pSMS: standard plus personalized SMS.

^c^S+VCS: standard plus videoconferencing support.

#### Videoconference Adherence

Participation in videoconferencing support was low, with 35.9% (37/103) of participants (in the S+VCS group) not attending any support sessions and only 18.4% (19/103) attending 7 or more out of 10 sessions.

### Preferences

Table 4 specifies the preferred mode of human support by group. A total of 88.7% (284/320) of participants indicated a valid human support preference. Almost half of participants within each group indicated a preference for combined support (ie, automated emails, personalized SMS, and VCS); however, the combined support alternative was not offered as part of the study. In comparison, the preference for solely S+VCS support was low across all the groups, with only between 7% to 8% choosing it from each group. Table 4 also indicates that 24% (22/91) in the S group, 27% (20/96) in the S+pSMS group, and 8% (8/97) in the S+VCS group received their preferred mode of support.

Secondary analysis was conducted to determine whether those who received their first preference for support mode scored better adherence or outcomes than those who did not receive their first choice. Participants (n=128) who chose the combined support as their first preference (an option not included in the comparative study) were excluded from the analysis, and the remaining participant data (n=156) were analyzed. Table 5 outlines the results between those who did not receive their first preference in human support mode and those who did. There was no significant difference between the 2 groups in either adherence or well-being measures.

**Table 4:** Preferences for modes of human support.

Support mode preference	S^a^ (n=91)	S+pSMS^b^ (n=96)	S+VCS^c^ (n=97)
Standard, n (%)	22 (24)	21 (22)	18 (19)
Standard plus SMS support, n (%)	25 (27)	26 (27)	23 (24)
Standard plus VCS^d^ support, n (%)	6 (6)	7 (7)	8 (8)
Standard plus SMS & VCS support, n (%)	38 (42)	42 (44)	48 (49)

^a^S: standard (automated emails only).

^b^S+pSMS: standard plus personalized SMS.

^c^S+VCS: standard plus videoconferencing support.

^d^VCS: videoconferencing support.

**Table 5:** The preferred human support mode’s effect on adherence and outcomes.

Variables			Preference				Between-group difference ** **		
			No (n=1000), mean (SD)		Yes (n=56), mean (SD)		*P* value		Cohen *d*
**Adherence**									
	Videos watched (out of 10)	6.1 (3.9)		6.5 (4.0)		.91		0.09	
	Challenge points (out of 100)	339.9 (352.2)		388.6 (385.9)		.27		0.13	
**Outcomes**									
	Mental health	8.4 (14.4)		9.8 (13.3)		.86		0.10	
	Vitality	9.5 (15.2)		10.6 (13.8)		.98		0.08	
	Depression	–1.3 (3.4)		–1.6 (2.5)		.09		0.10	
	Anxiety	–0.6 (1.9)		–1.2 (2.6)		.64		0.26	
	Stress	–1.0 (3.5)		–1.6 (2.8)		.55		0.19	
	Life satisfaction	2.2 (4.6)		1.9 (4.8)		.50		0.06	
	Flourishing	0.14 (.53)		0.21 (.53)		.47		0.13	

## Discussion

### Principal Results

Higher early dropout attrition occurred in the group which was allocated videoconferencing as a mode of human support. However, the mode of human support made no impact on attrition or adherence after the commencement of the intervention. Moreover, for participants who received their first preference in human support, compared to those who did not, no differences were observed in adherence. Preference for VCS support was low, yet almost half of the participants indicated they would prefer all forms of human support, though this was not an option in this study.

In this study, after the initial email notified participants of their group allocation, a disproportionate number of participants allocated to the S+VCS group withdrew from the study, with a significant between-group difference (*P*=.009). The early dropout attrition may have been influenced by dissatisfaction with the assigned support mode. Figure 1 shows that, in the S+VCS group, 57 participants either requested withdrawal or failed to register compared to 36 in the email group (S), and 28 in the S+pSMS group. Of the 24 participants who requested withdrawal from the S+VCS group, 10 stated that time was a factor, 8 provided no specific reason, 4 stated personal illness, 1 mentioned work commitments, and 1 participant stated they did not want to be involved in videoconferencing.

No differences in adherence between the groups were detected; and as documented in a previous report [39], we observed no differences between the groups in the well-being measures either. However, in observing that almost half (150/320, 46.9%) of the entire cohort were 100% adherent (ie, viewed all 10 videos), we conducted stratified analysis to compare well-being measures of those who were fully adherent (n=150) with those who were not (n=170). Significantly greater improvements were observed in the fully adherent group for life satisfaction (*P*=.011; *d*=0.15*)* and flourishing scores (*P*=.012; *d*=0.15), yet effect sizes were small.

While the primary aim of human support is usually to foster greater adherence [14,45,46], support did not influence adherence behavior for the participants in this study. While earlier research among clinical cohorts have indicated that supported interventions yielded better adherence and outcomes [29,47,48], numerous studies have also shown that human support made no difference [31,32,34,49-53]. Considering the extensive repertoire of previously identified factors affecting adherence, it is plausible that, in this nonclinical group, a wide range of variants may have influenced adherence at the individual level [14], reducing the potential impact of human support as a single factor. Therefore, exploring participants’ perceptions regarding influences on adherence is an important topic for further investigation.

The S+VCS was the least preferred support mode (7%), and low VCS attendance reflected the low preference for this type of support. Exploring reasons for the disinterest in videoconferencing as a mode of support may be a topic for further research, as videoconferencing has been used successfully in other group contexts [54-58] and has proven to be useful so long as technical support was provided [54]. Albeit, in a recent German study, 64% of patients were resistant to videoconferencing as a method of communication with health professionals. Notably, less than 1% reported previous experience with its use [59].

The S+VCS group allocation required an extra time commitment on behalf of participants compared to the other groups. Both email and SMS support required no effort by the participant—support was “pushed” to devices [7]. Conversely, videoconference support attempted to “pull” participants to an extra event, requiring effort and time to gain benefit from the support offered. Conceivably, the time and energy required to engage with that mode of support, loss of anonymity, technological barriers, unfamiliarity with videoconferencing software, or concerns about the group interaction may have been a barrier that facilitated significantly greater dropout. Additionally, further research should investigate if the preference for S+VCS would have been higher if support had been provided on an individual rather than a group basis.

For participants remaining in the S+VCS group, many demonstrated low engagement with the videoconferencing support. Consequently, by not engaging in the VCS support, they experienced a similar level of support to those in the S group (ie, automated emails only). This hindered the ability to draw meaningful between-group comparisons regarding the influence of human support offered through video conferencing on adherence.

Receiving their first preference in human support mode did not translate to participants reporting better adherence or outcomes in this study. We were unable to locate comparable studies for nonclinical groups. However, previous research among clinical cohorts has demonstrated that receiving support preferences may impact patient perceptions about the usefulness of an intervention [60] and improve adherence [28,61]. While a meta-analysis revealed that patients receiving their preferences demonstrated improved treatment satisfaction, adherence, and outcomes with moderate effect sizes [28], other research found that receiving the preferred option does not always impact outcomes [61,62]. A 2019 study of patients with anxiety and depression compared adherence and outcomes when patients chose their preferred support [63]. Interestingly, 78% chose the maximal support option, regular weekly support, and just 22% chose optional support (ie, support by request only). Yet, both groups achieved similar improvements in anxiety and depression scores and there were no differences in adherence. Contact between participants and therapist was much less for those who chose optional support, suggesting that similar results can be achieved with less time and cost investment.

Although combined human support (ie, access to automated emails, SMS, and videoconferencing) was not offered in the study, it is interesting that almost half of the participants in every group chose this as their preferred support option. While the reasons for this are unclear, it is hypothesized that it is a common trait of human nature to want access to everything possible, even though the available options are not necessarily utilized or needed. Further research exploring participant perceptions regarding human support modes and preferences is warranted.

### Strengths and Limitations

The intervention and its implementation were supported by established theory. While the intervention itself was underpinned by the theory of planned behavior [64], the design components of the intervention for the web- and mobile app–based platforms were informed by the persuasive design model [15] and human support elements reflected principles of the supportive accountability model [6]. The use of a sole facilitator as a support person provided consistency in the participant’s experience with regards to technical assistance, messaging, and videoconference facilitation. The study attracted a large cohort with a broad range of ages (18-81 years); and adherence data was easily collected through the eLMS and mobile app, avoiding the possibility of human error. Despite the addition of human support elements, the administration of the intervention using the eLMS and the mobile app provided acceptable scalability, portability, and accessibility, demonstrating the potential for broad-based mental health promotion.

Several limitations should be noted. The study attracted mainly White, well-educated women. While this is often seen in digital health interventions [65,66], it limits the ability to generalize more widely. Data gathering relied on self-reporting, which may be subject to bias, and self-selection into the study may have resulted in a cohort skewed by factors such as technological ability and motivation to achieve better mental health. Bias may have been introduced by the disproportionate number of participants who withdrew from the S+VCS group after being notified of their group allocation, reducing the validity of the randomization process. In asking participants to rank human support preferences, analyses were limited by including a preference option in the questionnaire that was not included in the study (ie, combined support involving email, SMS, and VCS). Almost half of the participants ranked combined support as their first preference and were consequently excluded from further investigation, reducing the power of the analyses. Additionally, some factors that would have been useful for group comparison were not measured. For example, asking participants to indicate their level of engagement with SMS and emails, and collecting data regarding preference for use of the mobile app compared to the web-based experience, would have provided an indication of engagement with the human support offered.

### Conclusions

The findings of this study indicate that a web- and mobile app–based MHPI for a nonclinical cohort can be designed and implemented to maximize accessibility, scalability, and adherence without the additional cost of human support. While early dropout attrition may have been influenced by displeasure with allocated support, adherence to a 10-week MHPI for a healthy cohort was not impacted by differing modes of human support. Engagement with videoconference support was suboptimal, hindering the ability to draw meaningful between-group comparisons. However, SMS support demonstrated no added value compared to automated email support. Adherence was not impacted by participants receiving their first preference for support. Future research should explore participant perspectives on adherence behaviors.

## Appendix

Multimedia Appendix 1 Website and app screenshots.

Multimedia Appendix 2 Detailed intervention overview.

## Abbreviations

ANZCTR: Australian New Zealand Clinical Trials Registry
DASS: Depression, Anxiety and Stress Scales
eLMS: electronic learning management system
MHPI: mental health promotion intervention
RCT: randomized controlled trial
S: standard (automated emails only)
S+VCS: standard plus videoconferencing support
S+pSMS: standard plus personalized SMS
SF-36: Short Form Health Survey
SWLS: Satisfaction With Life Scale
VCS: videoconference support session
