# Supplementary material for: The Influence of Three Modes of Human Support on Attrition and Adherence to a Web- and Mobile App–Based Mental Health Promotion Intervention in a Nonclinical Cohort: Randomized Comparative Study
Source: J Med Internet Res. 2020 Sep 29;22(9):e19945. doi: 10.2196/19945 (PMC7556377; doi:10.2196/19945)
Supplement: Multimedia Appendix 1 [file jmir_v22i9e19945_app1.pdf]

These images portray features of the website e-learning management system and mobile app relating to the following article:

A Web- and Mobile-app-based Mental Health Promotion Intervention Comparing Email, SMS and Videoconferencing Support for a Healthy Cohort: A Randomized Comparative Study

Authors: Renfrew M, Morton D, Morton J, Hinze J, Beamish P, Przybylko G, Craig B.

Section 1:

The Website

Images of the website
(e-learning management system)

https://eliawellness.com/

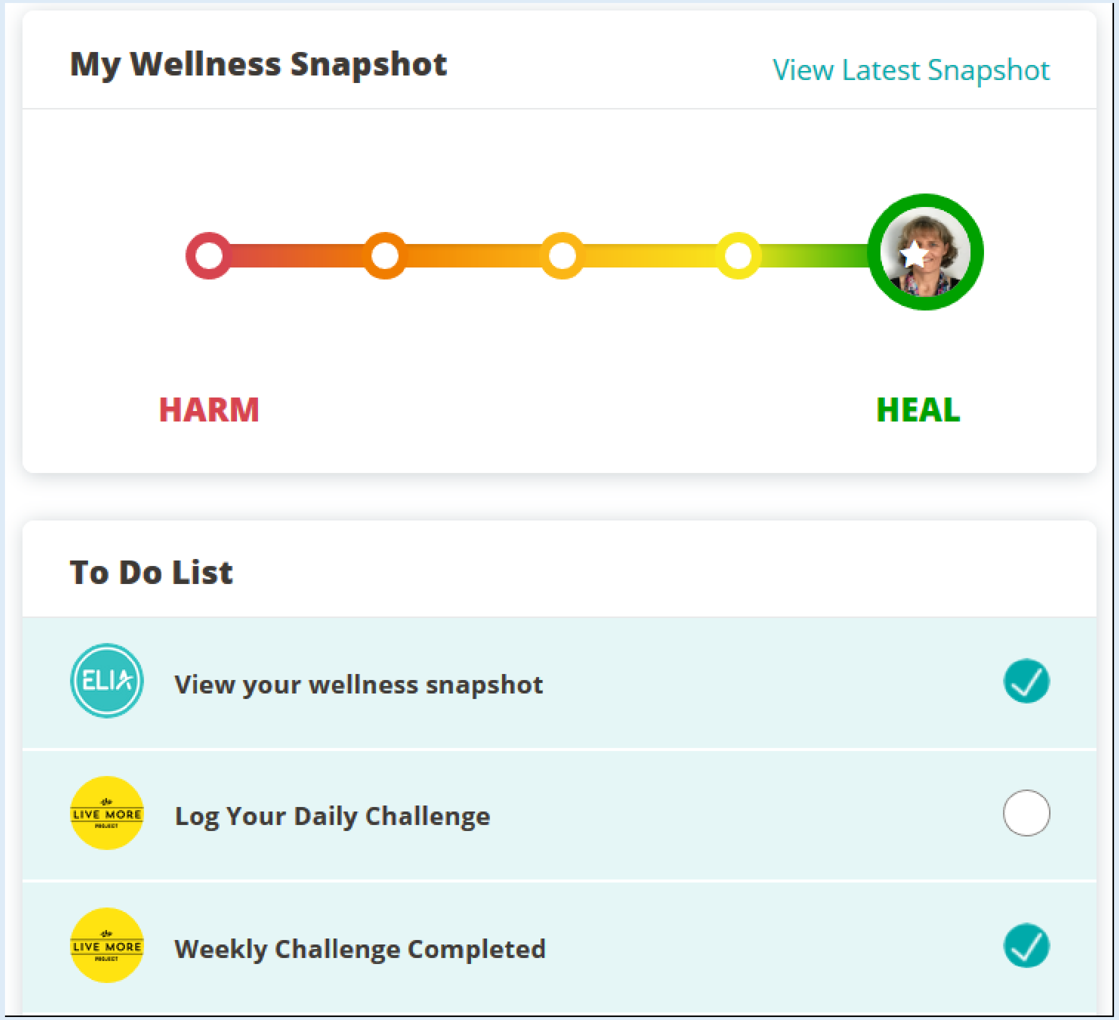

To Do List: An online "To Do" list (available on each personal dashboard) provided easily accessible links to complete the various components. Note: the "wellness snapshot" refers to the questionnaire administered at baseline and after completion of the program.

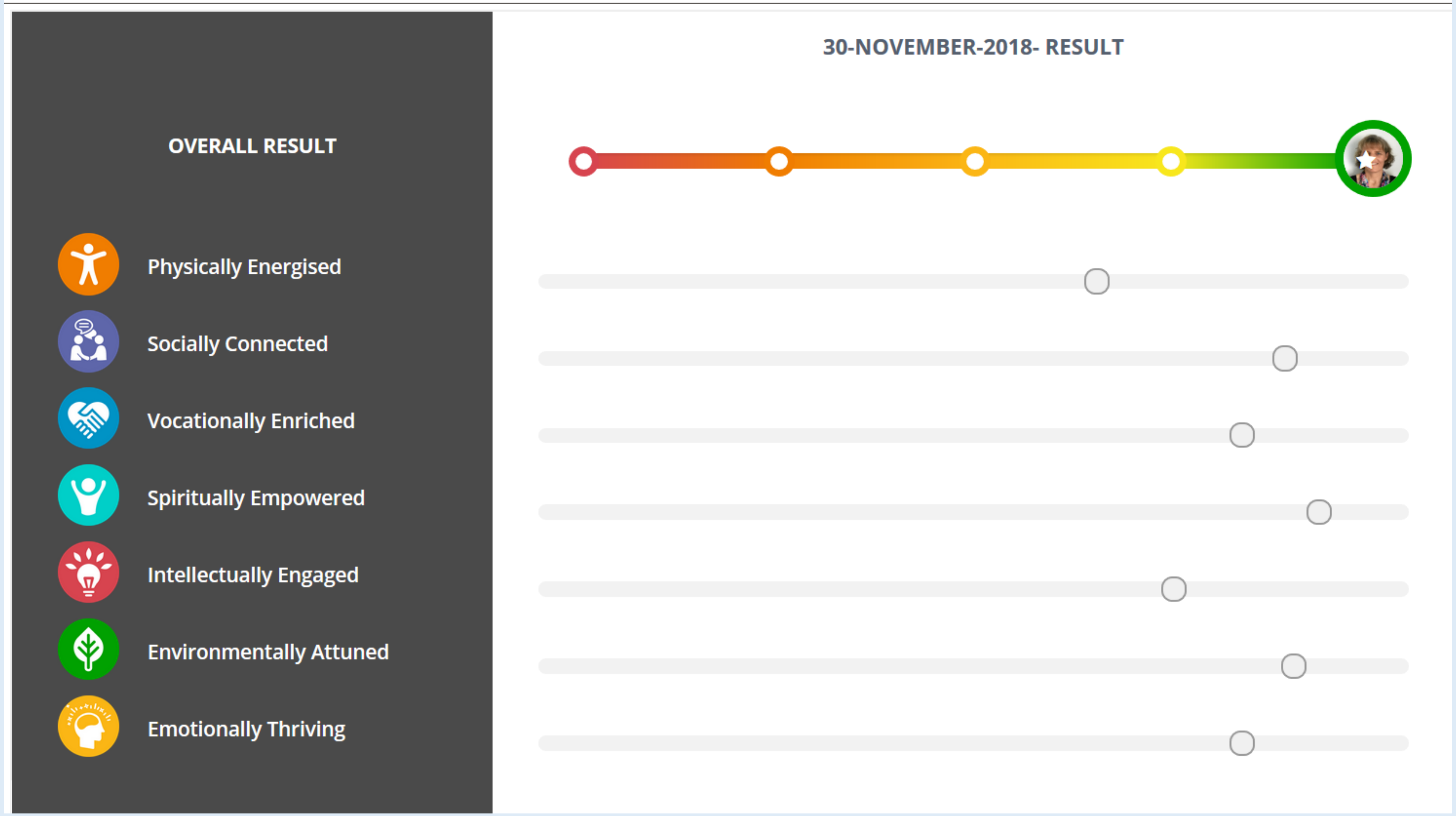

Overview of Wellness Snapshot Results: The "Wellness Snapshot" (i.e. questionnaire) provided a visual overview of where an individual was located on a continuum in seven different dimensions. A pie-chart view and percentage score out of 100 was also available.

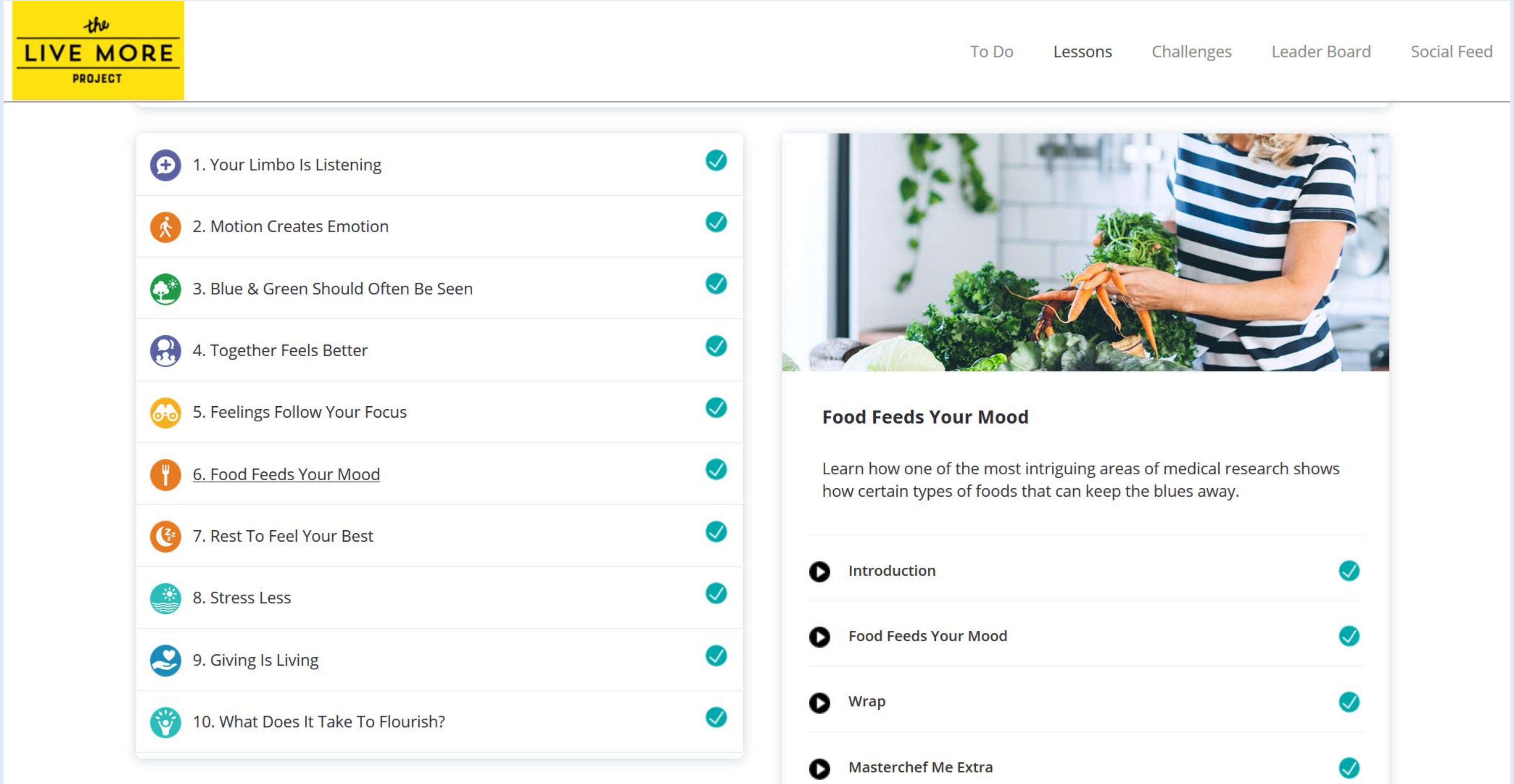

Sample View of Lesson 6 Content: Each lesson was divided into 3-4 short video presentations. Each new video segment was unlocked, on completion of the previous video. Segments were ticked off automatically as they were viewed.

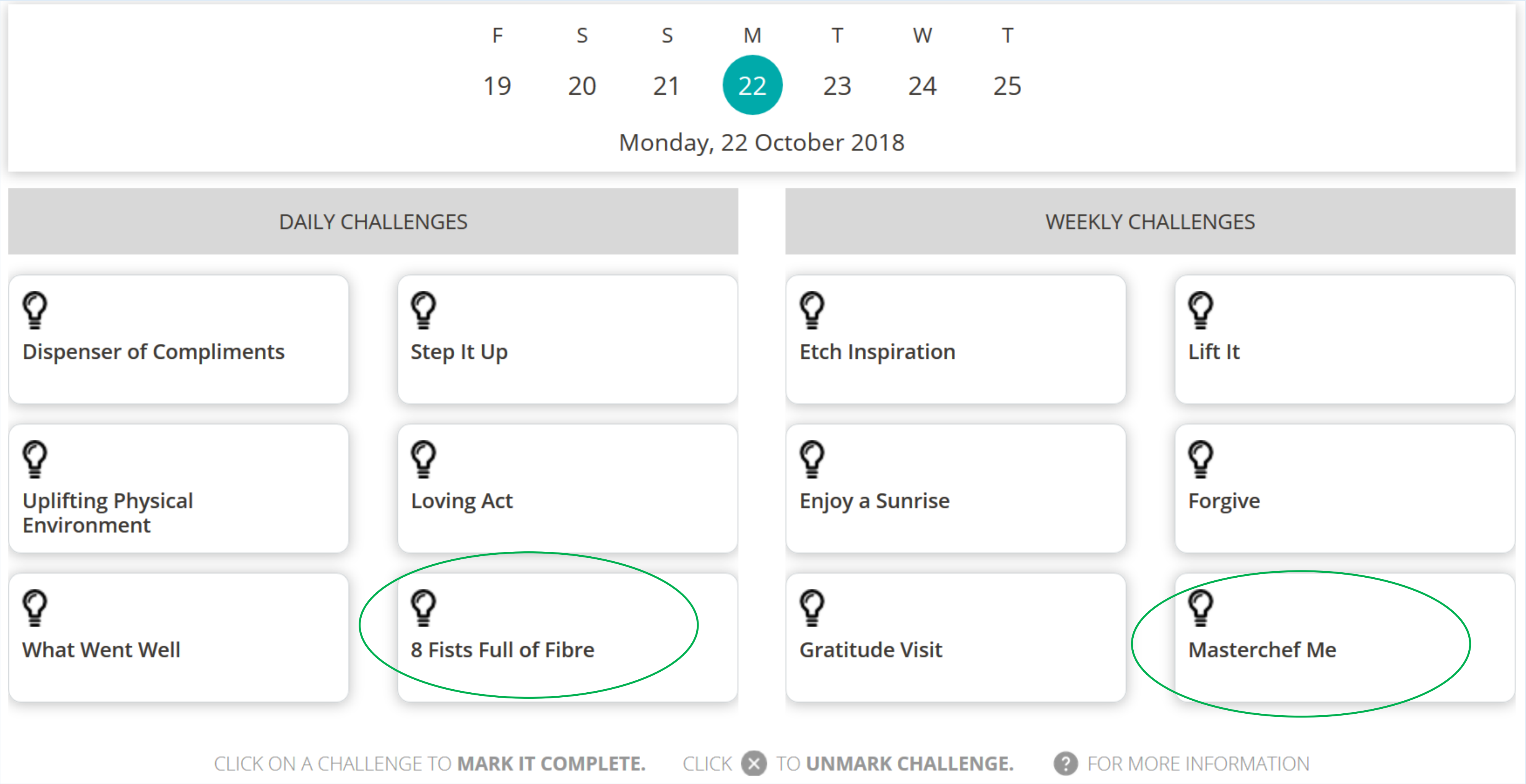

View of challenge icons for lessons 1-6: After each lesson, participants are provided with daily and weekly challenges. For example, for Lesson 6, participants were challenged to eat 8 fists full of fibre daily, and were asked to make 1 high-fibre plant based meal for the weekly challenge - “Masterchef Me”.

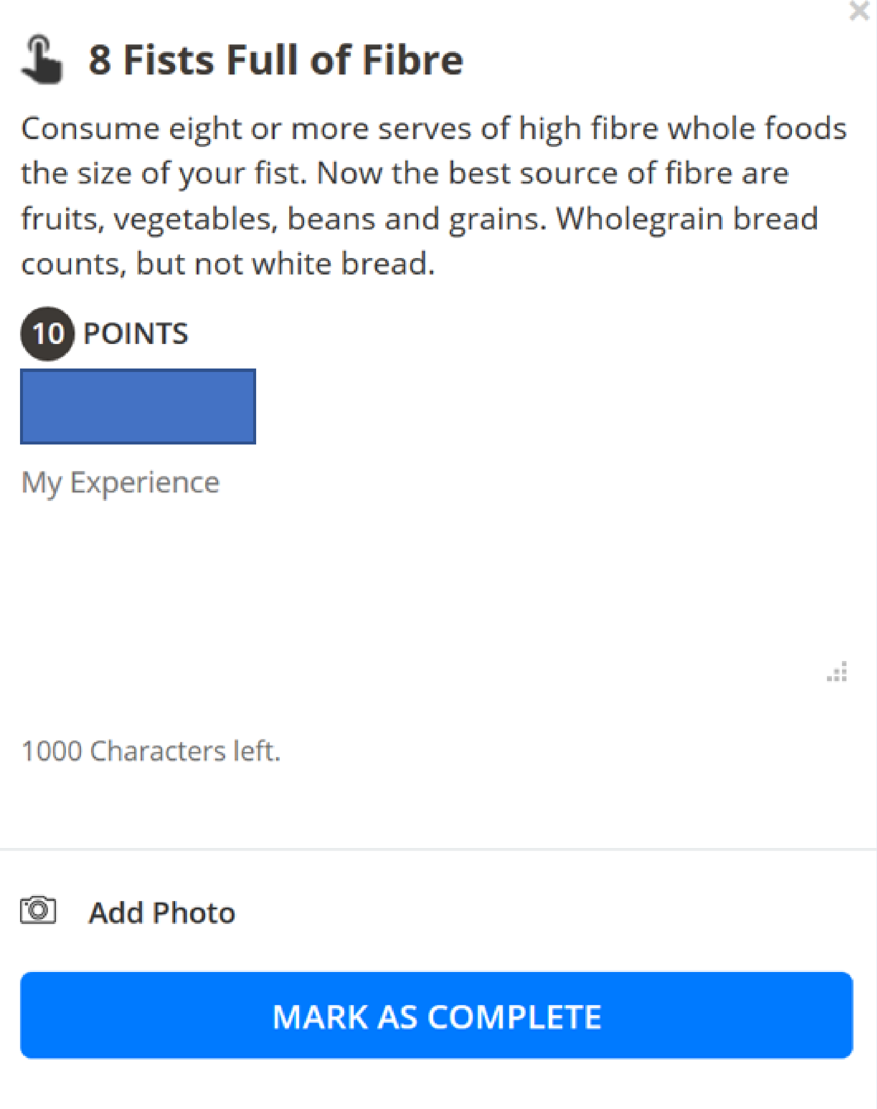

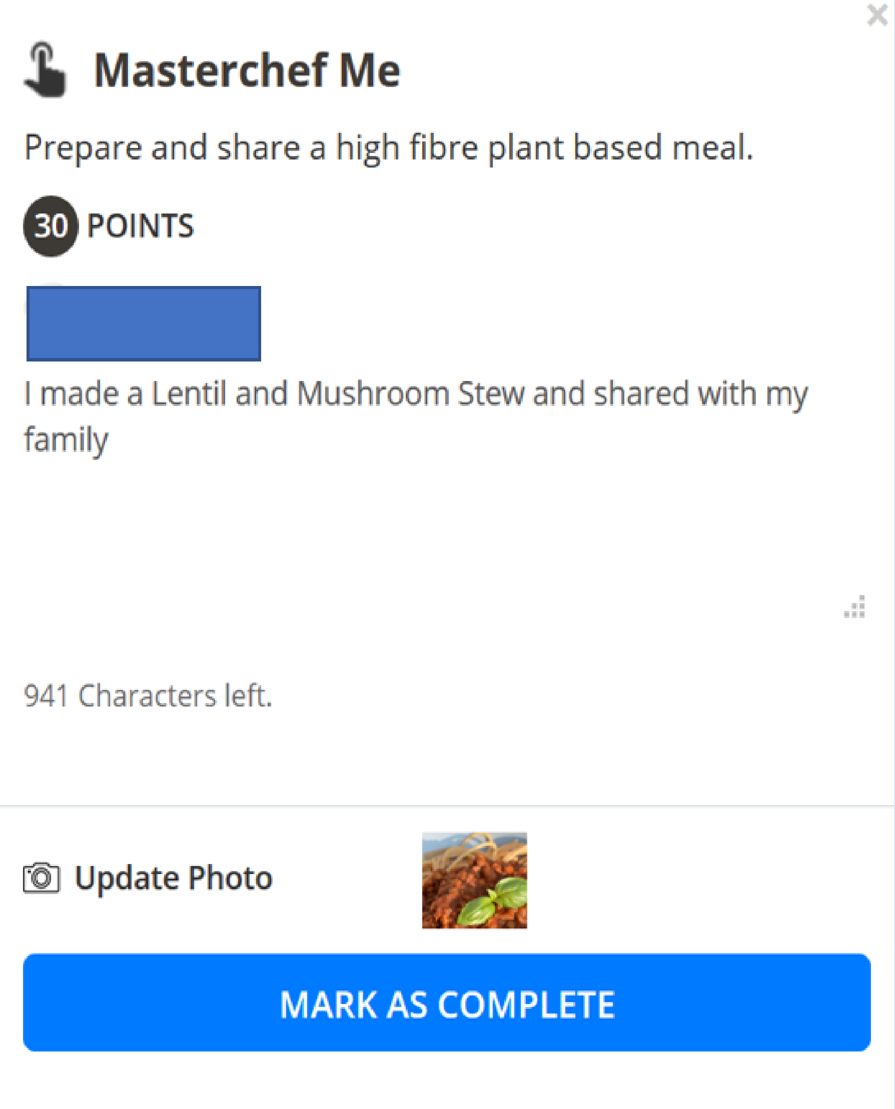

View of Lesson 6 Challenge Log Pages: When participants clicked on the challenge icon (previous screen) it opened up a screen to log daily challenges (possibility of 10 points scored daily) and weekly challenges (30 points scored per week). Name and photo ID has been obscured for privacy reasons

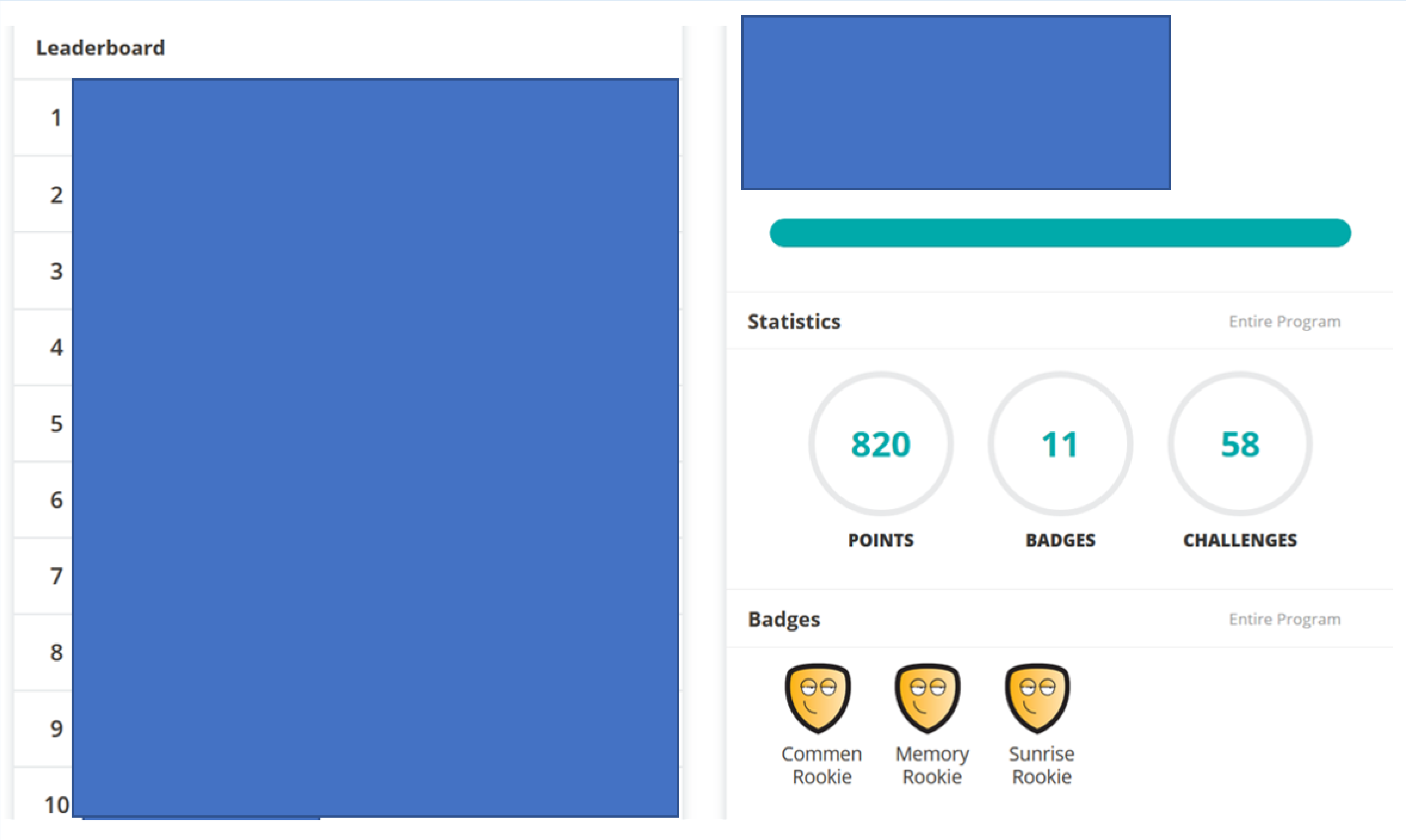

View of Leaderboard Names and photos have been blocked to protect privacy. Participants could compare their scores with others and earn badges for engagement

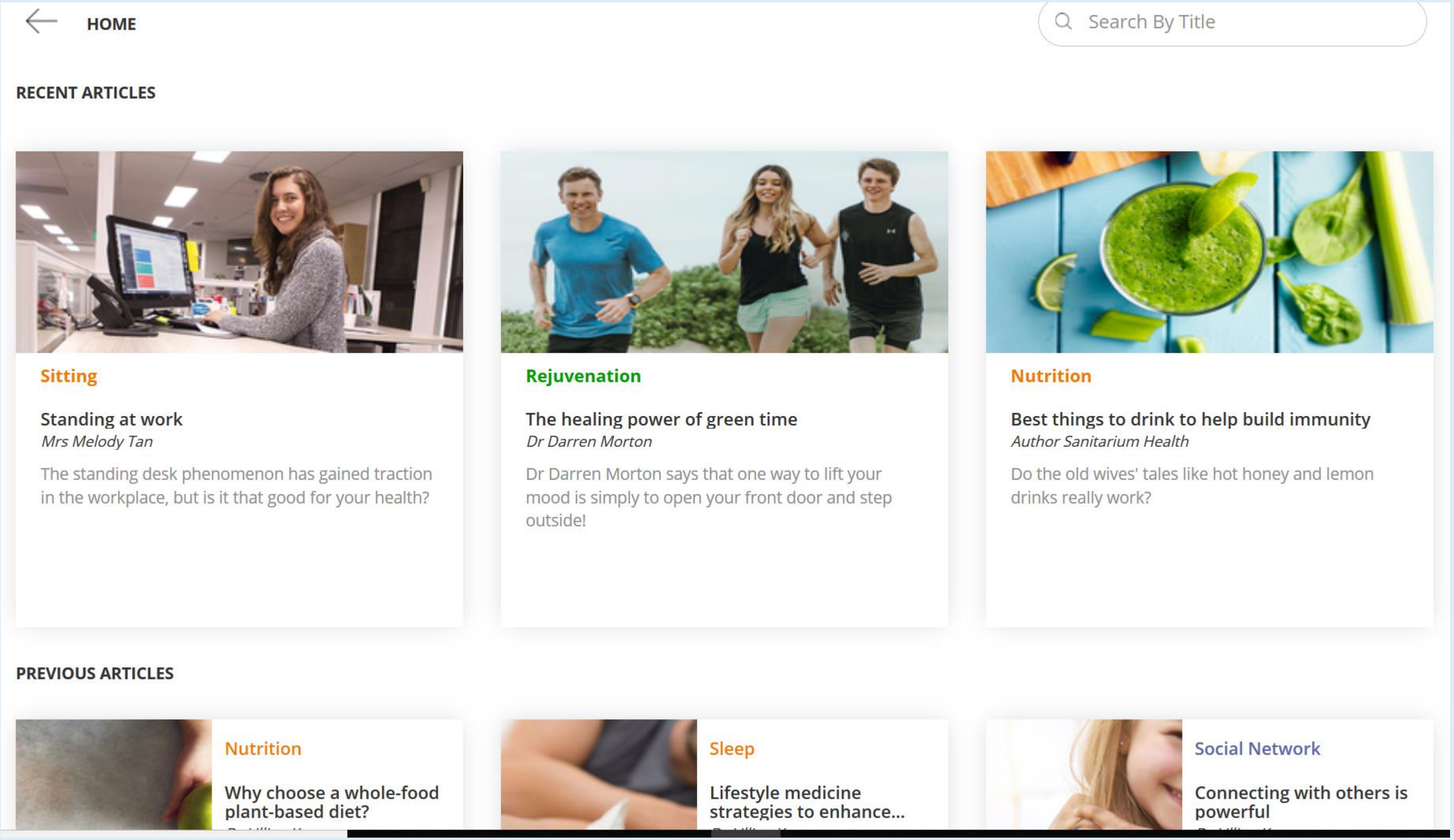

Extra articles on various topics: Participants could read extra information on topics covered

Section 2:

The App

The following images are from the mobile app called “mywellness” – available on the “App Store” and “Google Play”

https://apps.apple.com/us/app/mywellness/id1434379771?ls=1

https://play.google.com/store/apps/details?id=com.spd.eliawellness

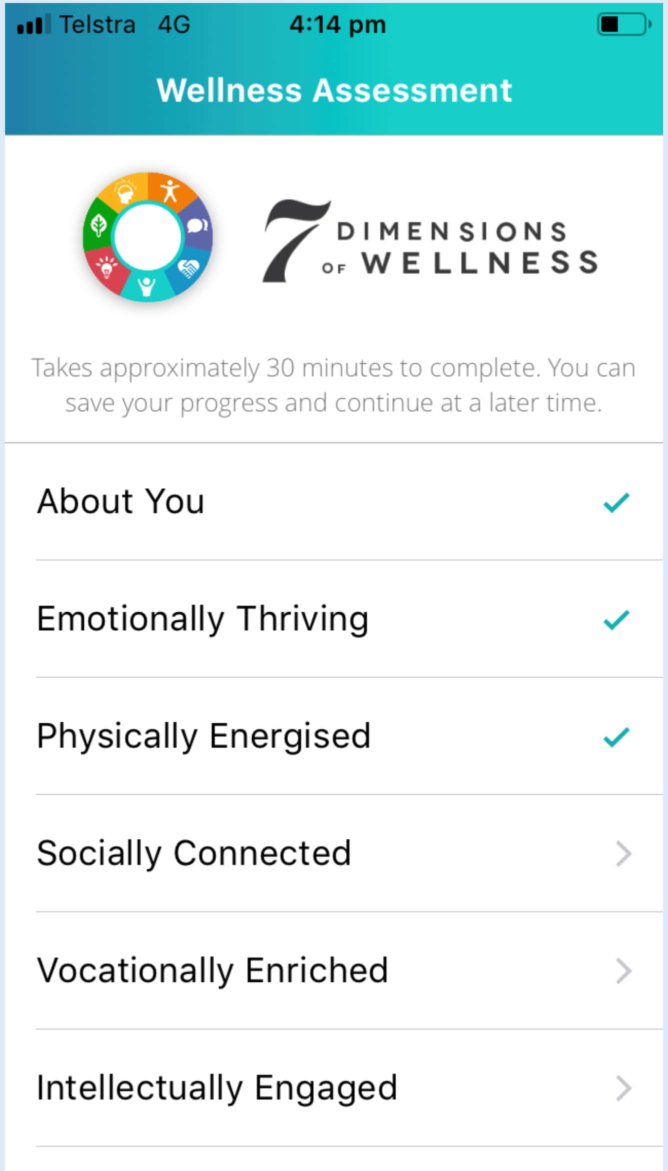

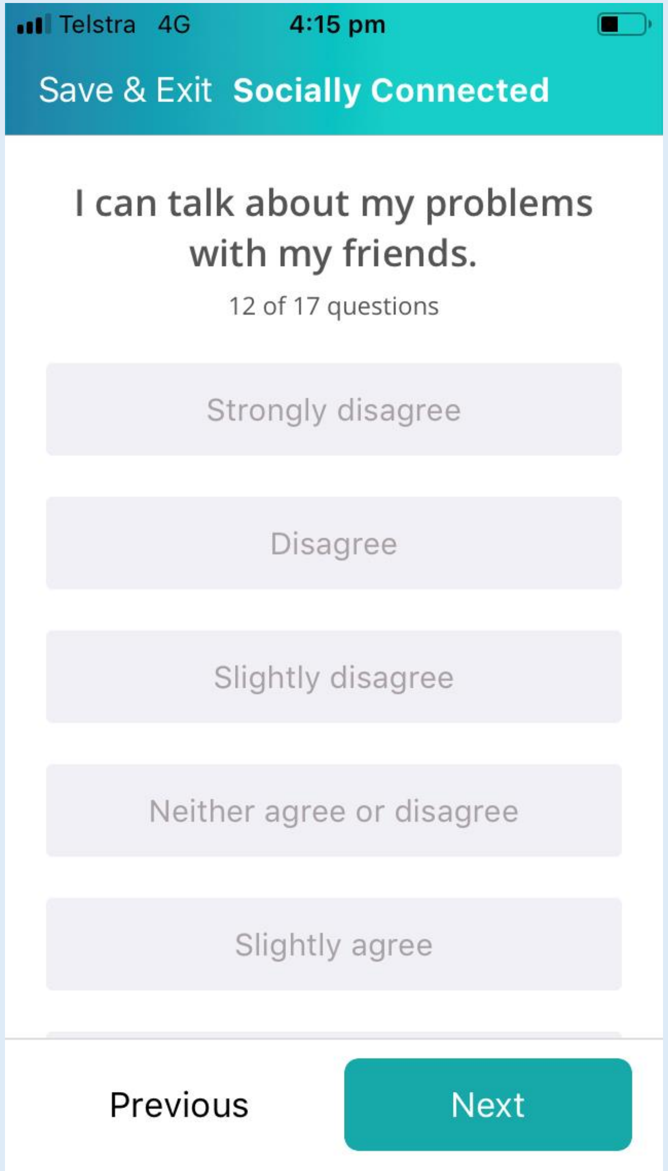

Questionnaire: Once a participant tapped the answer to the question on the screen, the screen automatically moved to the next question

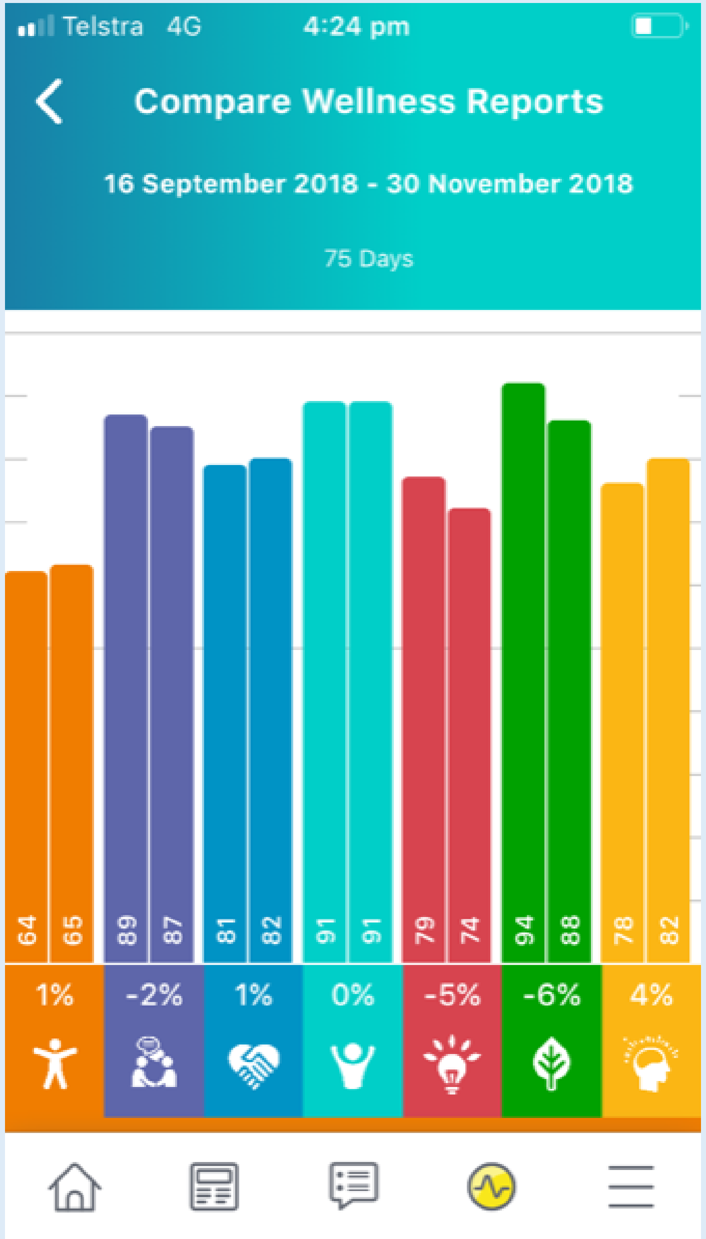

Comparison of Questionnaire Results – Pre & Post: Participants could compare their results before and after the intervention

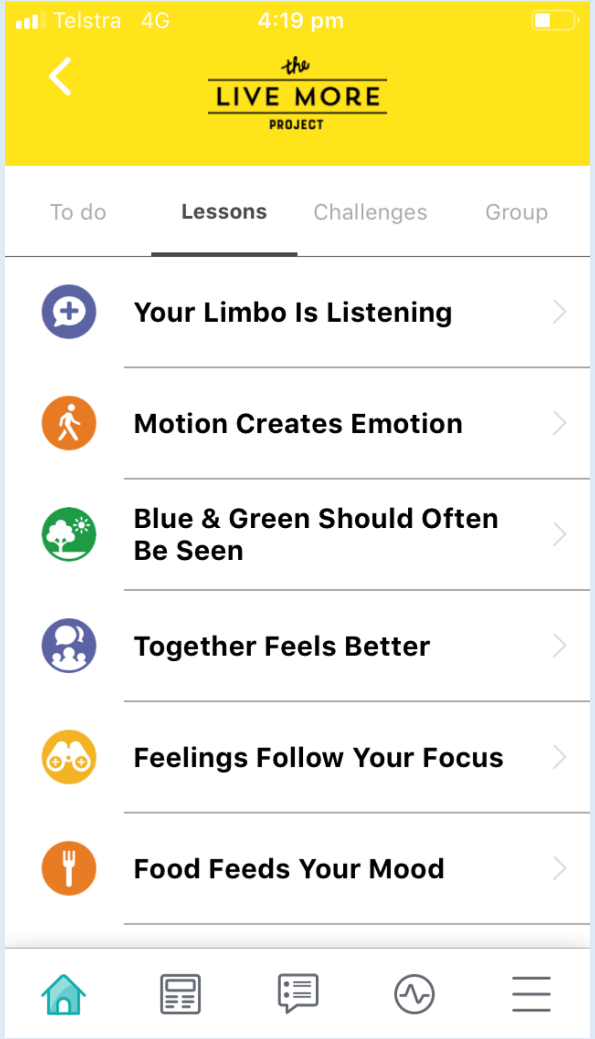

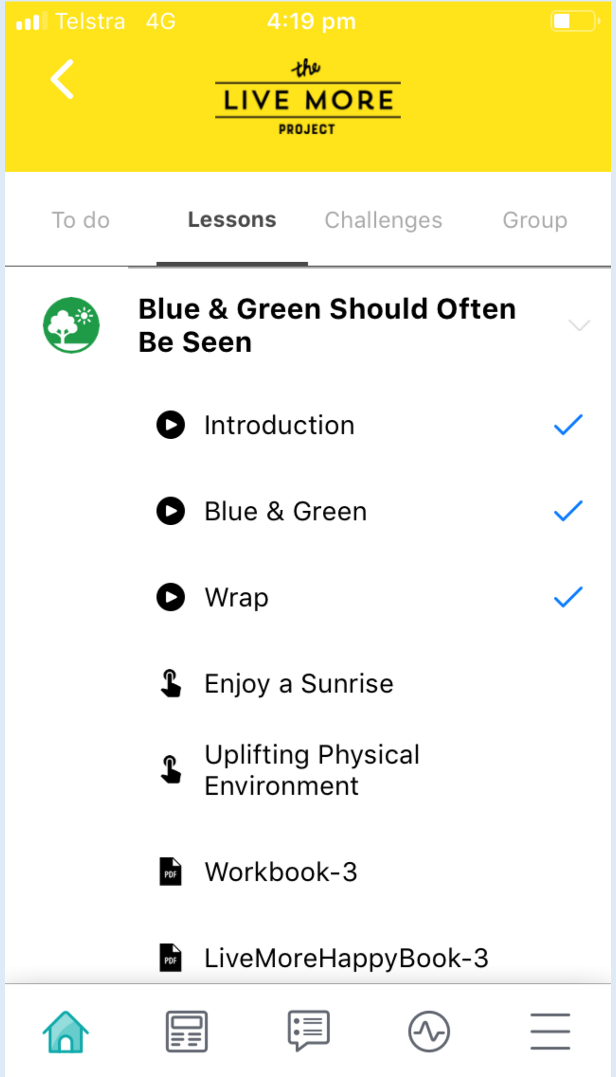

Video content on the mobile app: Participants watched the video content and could download a workbook and a chapter of the accompanying text-book book “Live More Happy” (Morton, 2018) from the Lessons page on the app (or the website).

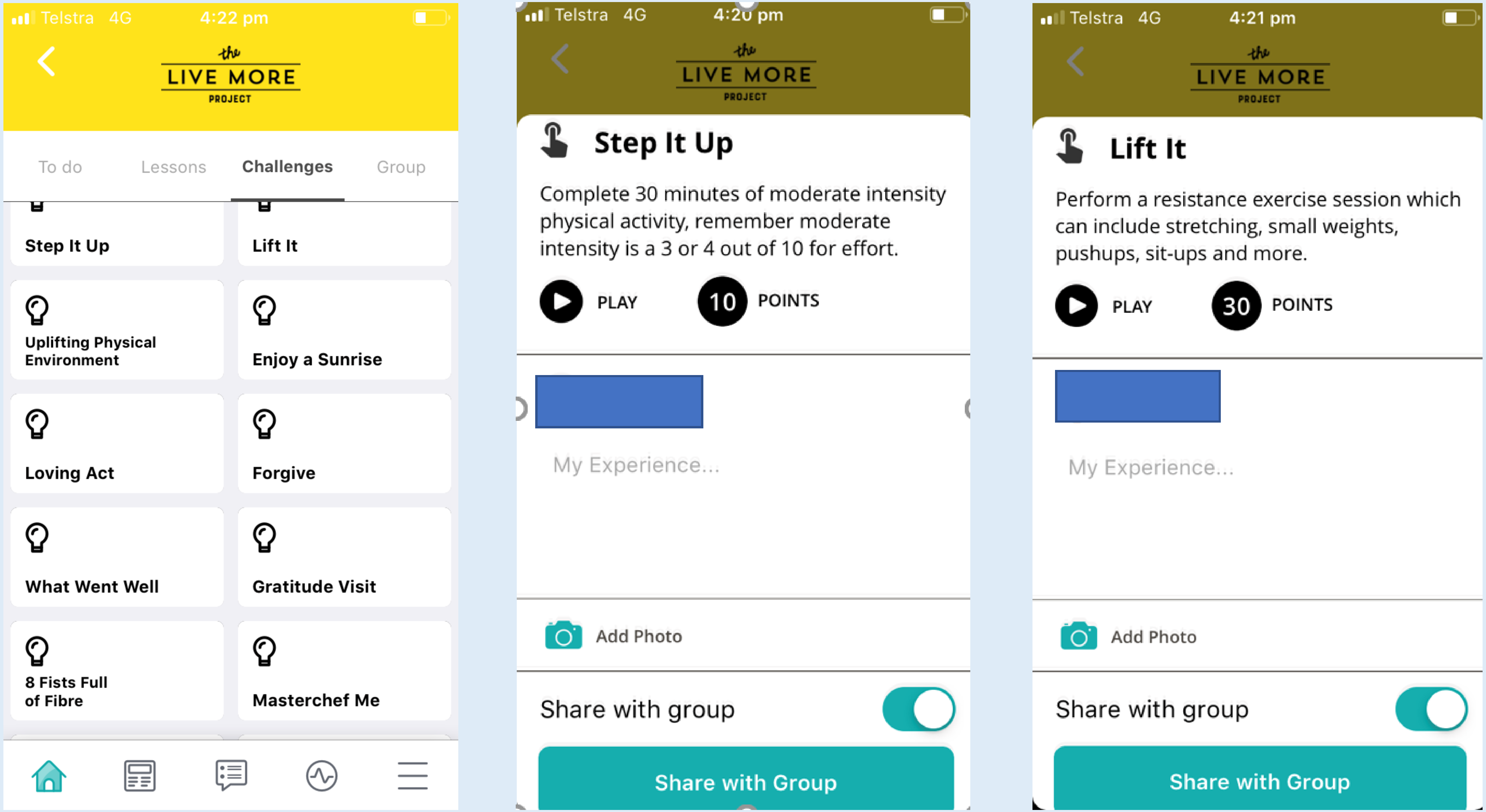

Challenges on the App: Participants could click on the icon and then record challenge activity while ‘on the go’.

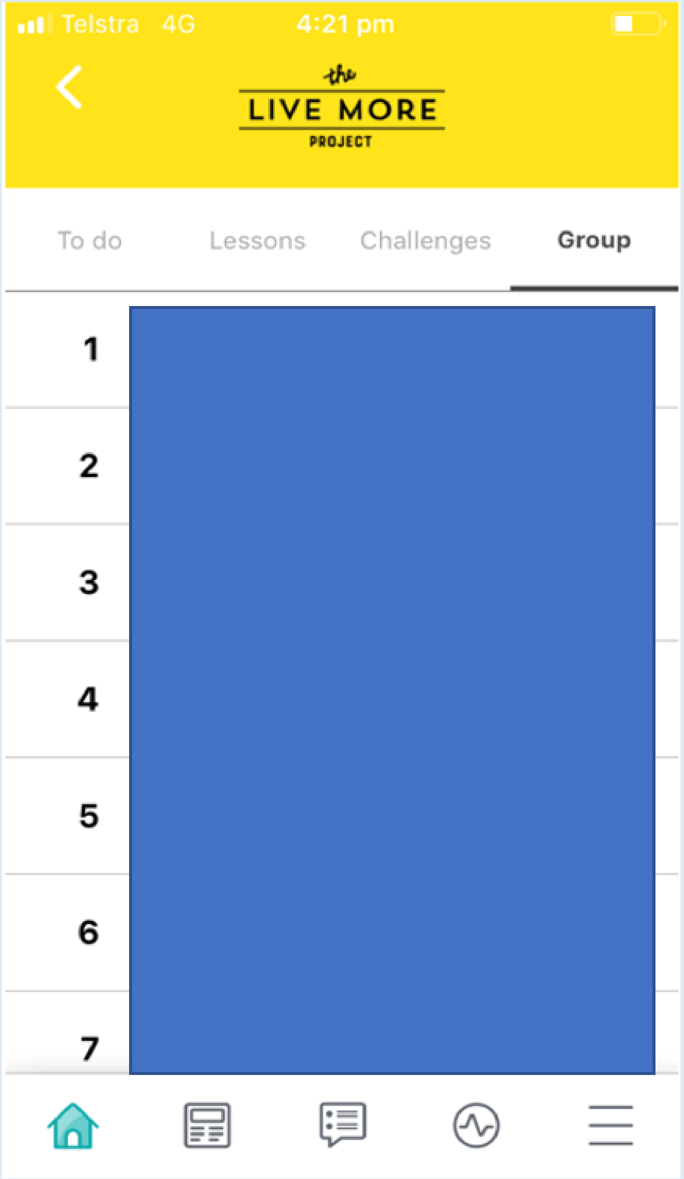

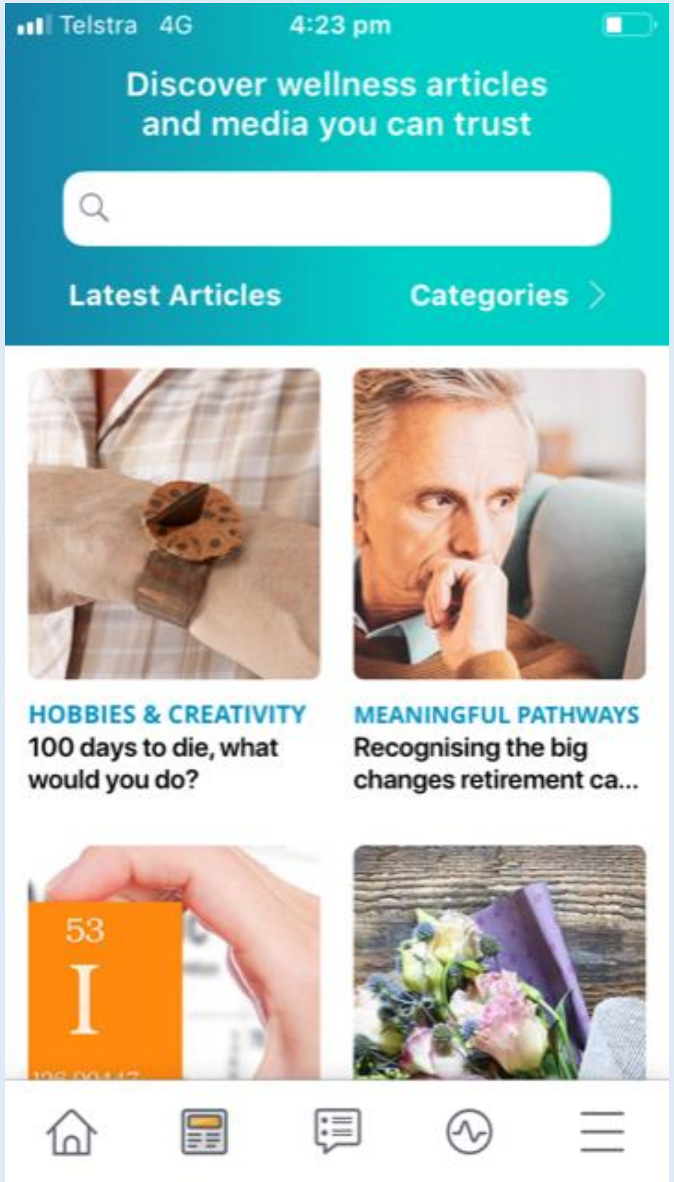

Leaderboard and extra articles: Names and photos have been removed for privacy. Participants could compare their progress with others on the leaderboard and read articles of interest related to the topics of the program.

References

Morton D, Live more happy : scientifically proven ways to lift your mood and your life. Warburton, Vic.: Signs Publishing Company; 2018; ISBN:9781925044720.
