# Supplementary material for: The Influence of Three Modes of Human Support on Attrition and Adherence to a Web- and Mobile App–Based Mental Health Promotion Intervention in a Nonclinical Cohort: Randomized Comparative Study
Source: J Med Internet Res. 2020 Sep 29;22(9):e19945. doi: 10.2196/19945 (PMC7556377; doi:10.2196/19945)
Supplement: Multimedia Appendix 2 [file jmir_v22i9e19945_app2.pdf]

Intervention Overview

Week	Topic	Overview	Daily Challenge	Weekly Challenge
1	**S**peak Positively (Your Limbo is Listening)	• Limbic System introduction – the "emotional brain".<br>• Limbic System is 'wired' to language area of brain.<br>• References [1, 2]	Offer a genuine compliment.	Memorise an inspirational text or saying.
2	**M**ove Dynamically (Motion Creates Emotion)	• Proprioceptors (nerve cells that detect movement) pass through the Limbic System.<br>• Movement (even just 10 minutes) improves mood.<br>• References [3-6]	Complete 30 minutes of moderate-intensity activity.	Perform resistance exercises once during week (exercises demonstrated on video).
3	**I**mmerse in an Uplifting Natural Environment (Blue and Green Should Often be Seen)	• The Limbic System receives messages from all the senses.<br>• The Limbic System likes blue and green spaces (i.e. natural settings).<br>• The Limbic System needs about 30 minutes of 10,000 LUX of light daily.<br>• References [7-9]	Immerse in an uplifting natural environment for 30 minutes daily.	Watch a sunrise from an appealing blue or green location.
4	**I**mmerse in a Positive Social Environment (Together Feels Better)	• Limbic systems communicate.<br>• Create positive social environments by making new friends or strengthening existing relationships.<br>• Forgiveness<br>• References [10-12]	Daily, do something intentional to show a friend or family member they are loved (use their love language).	Give up your right to hurt someone who has hurt you (Act of Forgiveness).
5	**L**ook to the Positive (Feelings Follow Your Focus)	• 'Emotional Brain' is wired to 'Thinking Brain'.<br>• What you focus on affects how you feel.<br>• Upward or downward spirals.<br>• References [13-17]	Write down three things that went well today "What Went Well?".	Gratitude visit – identify someone you are grateful to, write a gratitude letter to them, deliver and read in person, if possible.
6	**E**at Nutritiously (Food Feeds Your Mood)	• Gut bacteria linked to mood.<br>• Feed gut bacteria high fibre diet.<br>• Plant based foods are high fibre.<br>• Eat a wide variety of fruit, vegetables, legumes, grains.<br>• References [18-21]	Eat eight fists full of fibre daily.	Prepare and share a high-fibre plant based meal with one or more friends.
7	**R**est – Sleep (Rest to Feel Your Best)	• Sleep is fundamental for feeling upbeat (7-8 hours optimal).	Spend 8 hours in bed every night.	Spend an evening by firelight.

		• Blue light vs. yellow/orange light.<br>• Caffeine, lack of physical activity and blue light – deprived sleep.<br>• References [22, 23]		
8	**R**est – from Stress (Stress Less)	• SMILERS strategies 'open the valves'.<br>• Physical activity, practicing mindfulness, laughing, rest day.<br>• References [24-27]	Fifteen minutes 'sit in silence' mindful activity.	Take a 'guilt-free' day off.
9	**S**erve others (Giving is Living)	• Contributing/serving is emotionally uplifting.<br>• Serve sustainably using signature strengths.<br>• References [28-30]	Perform one or more random acts of kindness each day.	Use your significant strength to perform a significant act of service.
10	What Does it Take to Flourish?	• Five areas to flourish: PEARM – Positive emotions, engagement, achievement, relationships, meaning.<br>• References [29]	Spend time engaging in something you enjoy.	Create a list of goals and an action plan to achieve them.

References

1. Carrere, S., et al., *Predicting marital stability and divorce in newlywed couples.* Journal of Family Psychology, 2000. **14**(1): p. 42.
2. Losada, M., *The complex dynamics of high performance teams.* Mathematical and Computer Modelling, 1999. **30**(9): p. 179-192.
3. Nair, S., et al., *Do slumped and upright postures affect stress responses? A randomized trial.* Health Psychol, 2015. **34**(6): p. 632-41.
4. Josefsson, T., Lindwall, M., and Archer, T., *Physical exercise intervention in depressive disorders: Meta-analysis and systematic review.* Scandinavian journal of medicine & science in sports, 2014. **24**(2): p. 259-272.
5. Lathia, N., et al., *Happier people live more active lives: Using smartphones to link happiness and physical activity.* PloS one, 2017. **12**(1): p. e0160589.
6. Richards, J., et al., *Don't worry, be happy: cross-sectional associations between physical activity and happiness in 15 European countries.* BMC Public Health, 2015. **15**(1): p. 53-53.
7. Barton, J. and Pretty, J., *What is the best dose of nature and green exercise for improving mental health? A multi-study analysis.* Environmental science & technology, 2010. **44**(10): p. 3947-3955.
8. Mantler, A. and Logan, A.C., *Natural environments and mental health.* Advances in Integrative Medicine, 2015. **2**(1): p. 5-12.
9. McMahan, E.A. and Estes, D., *The effect of contact with natural environments on positive and negative affect: A meta-analysis.* The Journal of Positive Psychology, 2015. **10**(6): p. 507-519.
10. Fowler, J.H. and Christakis, N.A., *Dynamic spread of happiness in a large social network: longitudinal analysis over 20 years in the Framingham Heart Study.* Bmj, 2008. **337**: p. a2338.

11. Toussaint, L.L., Worthington, E., and Williams, D.R., *Forgiveness and health*. 2015: Springer.
12. Toussaint, L., et al., *Effects of lifetime stress exposure on mental and physical health in young adulthood: How stress degrades and forgiveness protects health.* Journal of health psychology, 2016. **21**(6): p. 1004-1014.
13. Garland, E.L., et al., *Upward spirals of positive emotions counter downward spirals of negativity: Insights from the broaden-and-build theory and affective neuroscience on the treatment of emotion dysfunctions and deficits in psychopathology.* Clinical psychology review, 2010. **30**(7): p. 849-864.
14. Emmons, R.A. and McCullough, M.E., *Counting blessings versus burdens: an experimental investigation of gratitude and subjective well-being in daily life.* Journal of personality and social psychology, 2003. **84**(2): p. 377.
15. Froh, J.J., Sefick, W.J., and Emmons, R.A., *Counting blessings in early adolescents: An experimental study of gratitude and subjective well-being.* Journal of school psychology, 2008. **46**(2): p. 213-233.
16. Proyer, R.T., et al., *Positive psychology interventions in people aged 50–79 years: long-term effects of placebo-controlled online interventions on well-being and depression.* Aging & Mental Health, 2014. **18**(8): p. 997-1005.
17. Seligman, M.E.P., et al., *Positive Psychology Progress: Empirical Validation of Interventions.* American Psychologist, 2005. **60**(5): p. 410-421.
18. Blanchflower, D.G., Oswald, A.J., and Stewart-Brown, S., *Is psychological well-being linked to the consumption of fruit and vegetables?* Social Indicators Research, 2013. **114**(3): p. 785-801.
19. Jacka, F.N., et al., *A randomised controlled trial of dietary improvement for adults with major depression (the 'SMILES'trial).* BMC medicine, 2017. **15**(1): p. 23.
20. Quirk, S.E., et al., *The association between diet quality, dietary patterns and depression in adults: a systematic review.* BMC psychiatry, 2013. **13**(1): p. 175.
21. White, B.A., Horwath, C.C., and Conner, T.S., *Many apples a day keep the blues away–Daily experiences of negative and positive affect and food consumption in young adults.* British Journal of Health Psychology, 2013. **18**(4): p. 782-798.
22. Neckelmann, D., Mykletun, A., and Dahl, A.A., *Chronic insomnia as a risk factor for developing anxiety and depression.* Sleep, 2007. **30**(7): p. 873-880.
23. Bedrosian, T. and Nelson, R., *Influence of the modern light environment on mood.* Molecular psychiatry, 2013. **18**(7): p. 751.
24. Heber, E., et al., *The benefit of Web- and computer-based interventions for stress: A systematic review and meta-analysis.* J Med Internet Res, 2017. **19**(2): p. e32.
25. Keng, S.-L., Smoski, M.J., and Robins, C.J., *Effects of mindfulness on psychological health: A review of empirical studies.* Clinical psychology review, 2011. **31**(6): p. 1041-1056.
26. Cornelissen, G., et al., *Mapping of circaseptan and circadian changes in mood.* Scripta medica, 2005. **78**(2): p. 89.
27. Smith-Gabai, H. and Ludwig, F., *Observing the Jewish Sabbath: A meaningful restorative ritual for modern times.* Journal of Occupational Science, 2011. **18**(4): p. 347-355.
28. Buchanan, K.E. and Bardi, A., *Acts of kindness and acts of novelty affect life satisfaction.* The Journal of social psychology, 2010. **150**(3): p. 235-237.
29. Seligman, M.E.P., *Flourish : a visionary new understanding of happiness and well-being*. 2013, New York: Free Press.
30. Tkach, C.T., *Unlocking the treasury of human kindness: Enduring improvements in mood, happiness, and self-evaluations*. 2006, US: ProQuest Information & Learning.
